# Supplementary material for: Visiting Molecular Mimicry Once More: Pathogenicity, Virulence, and Autoimmunity
Source: Microorganisms. 2023 Jun 1;11(6):1472. doi: 10.3390/microorganisms11061472 (PMC10301193; doi:10.3390/microorganisms11061472)
Supplement: Supplementary file 1 [file microorganisms-11-01472-s001.zip › microorganisms-2264476-supplementary.pdf]

Supplementary Material

Table S1: Number of similar proteins between humans and different organisms.

Organism	Total of proteins	Low stringency			High stringency		
		E-value					
		**1^{-10}**	**1^{-50}**	**1^{-100}**	**1^{-10}**	**1^{-50}**	**1^{-100}**
H. sapiens	75,074						
Pathogenic organisms							
P. falciparum	5,456	2769 (50.75)	955 (17.50)	322 (5.90)	103 (1.89)	81 (1.48)	41 (0.75)
E. histolytica	7,959	3357 (42.18)	1170 (14.70)	399 (5.01)	35 (0.44)	32 (0.40)	19 (0.24)
G. intestinalis	4,466	2209 (49.46)	685 (15.34)	172 (3.85)	17 (0.38)	15 (0.34)	11 (0.25)
T. gondii	8,404	3311 (39.40)	1357 (16.15)	517 (6.15)	118 (1.40)	107 (1.27)	61 (0.73)
T. brucei brucei	8,587	3711 (43.22)	1386 (16.14)	469 (5.46)	72 (0.84)	70 (0.82)	47 (0.55)
T. cruzi	19,242	3858 (20.05)	1426 (7.41)	499 (2.59)	75 (0.39)	73 (0.38)	48 (0.25)
C. neoformans	6,743	4734 (70.21)	2091 (31.01)	835 (12.38)	179 (2.65)	136 (2.02)	79 (1.17)
B. malayi	9,409	7828 (83.2)	4296 (45.66)	1815 (19.29)	318 (3.38)	285 (3.03)	147 (1.56)
L. major	8,038	3627 (45.12)	1360 (16.92)	451 (5.61)	72 (0.90)	70 (0.87)	45 (0.56)
C. tetani	2,415	1115 (46.17)	278 (11.51)	68 (2.82)	0 (0)	0 (0)	0 (0)
C. botulinum	3,590	1220 (33.98)	320 (8.91)	78 (2.17)	0 (0)	0 (0)	0 (0)
C. perfringens	2,721	1116 (41.01)	302 (11.10)	83 (3.05)	0 (0)	0 (0)	0 (0)
N. meningitidis	2,001	837 (41.83)	264 (13.19)	74 (3.70)	0 (0)	0 (0)	0 (0)
N. gonorrhoeae	2,106	794 (37.70)	241 (11.44)	71 (3.37)	0 (0)	0 (0)	0 (0)
C. albicans	6,035	4322 (71.62)	1881 (31.17)	707 (11.71)	161 (2.67)	118 (1.96)	70 (1.16)
C. glabrata	5,200	4060 (78.08)	1740 (33.46)	669 (12.87)	135 (2.60)	96 (1.85)	59 (1.13)
C. tropicalis	6,226	4157 (66.77)	1794 (28.81)	682 (10.95)	132 (2.12)	98 (1.57)	58 (0.93)
T. vaginalis	50,190	3922 (7.81)	1144 (2.28)	355 (0.71)	63 (0.13)	42 (0.08)	32 (0.06)

S. liquefaciens	5,531	1299 (23.49)	389 (7.03)	124 (2.24)	2 (0.04)	1 (0.02)	1 (0.02)
S. odoriferae	5,121	1284 (25.07)	385 (7.52)	116 (2.27)	2 (0.04)	1 (0.02)	1 (0.02)
S. ficaria	4,652	1273 (27.36)	387 (8.32)	125 (2.69)	2 (0.04)	1 (0.02)	1 (0.02)
B. cereus	5,240	1487 (28.38)	392 (7.48)	109 (2.08)	0 (0)	0 (0)	0 (0)
B. subtilis	4,260	1429 (33.54)	401 (9.41)	104 (2.44)	0 (0)	0 (0)	0 (0)
B. licheniformis	4,164	1478 (35.49)	401 (9.63)	109 (2.62)	0 (0)	0 (0)	0 (0)
T. marneffei	10,448	4901 (46.91)	2192 (20.98)	895 (8.57)	154 (1.47)	128 (1.23)	68 (0.65)
Nonpathogenic organisms							
T. rubripes	51,078	16470 (32.24)	13161 (25.77)	9442 (18.49)	3843 (7.52)	3686 (7.22)	3063 (6.00)
A. thaliana	39,351	6000 (15.25)	2632 (6.69)	1024 (2.60)	205 (0.52)	155 (0.39)	89 (0.23)
C. elegans	26,714	9551 (35.75)	5242 (19.62)	2227 (8.34)	364 (1.36)	323 (1.21)	165 (0.62)
C. intestinalis	17,309	11562 (66.80)	7672 (44.32)	3834 (22.15)	673 (3.89)	607 (3.51)	383 (2.21)
D. melanogaster	22,049	10125 (45.92)	6523 (29.58)	3302 (14.98)	625 (2.83)	544 (2.47)	332 (1.51)
M. musculus	55,471	19259 (34.72)	17700 (31.91)	15110 (27.24)	15216 (27.43)	14683 (26.47)	13128 (26.67)
O. sativa	48,903	5943 (12.15)	2471 (5.05)	931 (1.90)	196 (0.40)	156 (0.32)	88 (0.18)
R. norvegicus	29,943	19029 (63.55)	17491 (58.41)	14834 (49.54)	14617 (48.82)	14131 (47.19)	12603 (42.09)
S. cerevisiae	6,049	4088 (67.58)	1770 (29.26)	698 (11.54)	135 (2.23)	96 (1.59)	60 (0.99)
S. castellii	5,565	4038 (72.56)	1732 (31.12)	673 (12.09)	128 (2.30)	90 (1.62)	53 (0.95)
C. tyrobutyricum	3,032	1154 (38.06)	297 (9.80)	77 (2.54)	0 (0)	0 (0)	0 (0)
C. acetobutylicum	3,847	1163 (30.23)	309 (8.03)	75 (1.95)	0 (0)	0 (0)	0 (0)
C. chromiireducens	4,771	1282 (26.87)	317 (6.64)	90 (1.89)	1 (0.02)	1 (0.02)	1 (0.02)
N. sicca	3646	951 (26.08)	267 (7.32)	75 (2.06)	1 (0.03)	1 (0.03)	0 (0)
N. elongata	2073	835 (40.28)	266 (12.83)	75 (3.62)	0 (0)	0 (0)	0 (0)
N. bacilliformis	2,966	851 (28.69)	249 (8.40)	70 (2.36)	1 (0.03)	0 (0)	0 (0)

E. invadens	9,857	2967	984	322	23	22	14
		(30.10)	(9.98)	(3.27)	(0.23)	(0.22)	(0.14)
S. symbiotica	2,157	771	231	69	2	1	1
		(35.74)	(10.71)	(3.20)	(0.09)	(0.05)	(0.05)
B. pseudofirmus	4,310	1330	385	106	0	0	0
		(30.86)	(8.93)	(2.46)	(0)	(0)	(0)
P. roqueforti	12,315	5015	2167	868	169	142	69
		(40.72)	(17.60)	(7.05)	(1.37)	(1.15)	(0.56)
B. fuckeliana	11,022	4986	2201	894	163	150	75
		(45.24)	(19.97)	(8.11)	(1.48)	(1.36)	(0.68)

Low stringency = minimum percent coverage of 30% and minimum percent identity of 10%; High stringency = minimum percent coverage of 70% and minimum percent identity of 70%. () = Percentage of similar proteins to the human proteome calculated from the count of similar hits in relation to the total number of proteins of each species.

Table S2: Percentage of similar proteins between mice and different pathogenic organisms

**Organism**	**Total of proteins**	**Pathogenic potential**	**Low stringency**			**High stringency**		
			**E-value**					
			1^{-10}	1^{-50}	1^{-100}	1^{-10}	1^{-50}	1^{-100}
M. musculus	55,471							
F. tularensis	1,528	1.6×10^{0}	59.55	17.08	4.91	0.00	0.00	0.00
			(910)	(261)	(75)	(0)	(0)	(0)
B. anthracis	5,493	1.2×10^{0}	28.02	7.37	2.09	0.00	0.00	0.00
			(1539)	(405)	(115)	(0)	(0)	(0)
B. suis	1,091	8.3×10^{-1}	38.41	8.89	2.11	0.18	0.18	0.09
			(419)	(97)	(23)	(2)	(2)	(1)
T. gondii	8,404	2.1×10^{-1}	40.04	16.54	6.16	1.50	1.36	0.69
			(3365)	(1390)	(518)	(126)	(114)	(58)
C. immitis	9,712	1.9×10^{-1}	50.85	22.21	9.12	2.00	1.54	0.70
			(4939)	(2157)	(886)	(886)	(194)	(68)
K. pneumoniae	5,728	1.7×10^{-1}	22.64	6.72	2.09	0.03	0.03	0.02
			(1297)	(385)	(120)	(2)	(2)	(1)
S. pneumoniae	2,030	1.1×10^{-1}	49.51	13.99	2.91	0.00	0.00	0.00
			(1005)	(284)	(59)	(0)	(0)	(0)
Y. pestis	3,909	8.5×10^{-2}	28.27	9.24	3.04	0.05	0.03	0.03
			(1105)	(361)	(119)	(2)	(1)	(1)
C. neoformans	6,743	6.2×10^{-2}	70.64	30.70	12.35	2.71	1.93	1.08
			(4763)	(2070)	(833)	(183)	(130)	(73)
V. vulnificus	4,990	4.2×10^{-2}	28.78	7.68	2.40	0.02	0.00	0.00
			(1436)	(383)	(120)	(1)	(0)	(0)
E. coli k12	4,448	3.2×10^{-3}	27.92	8.36	2.68	0.04	0.04	0.02
			(1242)	(372)	(119)	(2)	(2)	(1)
C. albicans	6,035	4.8×10^{-4}	72.41	31.17	11.53	2.55	1.81	1.03
			(4370)	(1881)	(696)	(154)	(109)	(62)

A. fumigatus	9,647	3.0×10^{-5}	52.24 (5040)	22.69 (2189)	9.43 (910)	1.93 (186)	1.67 (161)	0.67 (65)
S. agalactiae	2,105	5.0×10^{-5}	47.03 (990)	11.78 (248)	2.85 (60)	0.00 (0)	0.00 (0)	0.00 (0)
L. monocytogenes	2,844	1.3×10^{-5}	42.83 (1218)	10.79 (307)	2.50 (71)	0.00 (0)	0.00 (0)	0.00 (0)
N. asteroides	6,459	3.7×10^{-6}	25.17 (1626)	6.38 (412)	1.30 (84)	0.00 (0)	0.00 (0)	0.00 (0)
N. fowleri	13,596	1.1×10^{-6}	40.77 (5543)	17.56 (2388)	6.62 (900)	0.75 (102)	0.67 (91)	0.37 (50)
B. cereus	5,240	3.2×10^{-7}	22.10 (1158)	7.82 (410)	2.12 (111)	0.00 (0)	0.00 (0)	0.00 (0)
S. saprophyticus	2,404	1.2×10^{-7}	47.80 (1149)	12.56 (302)	2.91 (70)	0.00 (0)	0.00 (0)	0.00 (0)
P. aeruginosa	5,564	6.3×10^{-8}	25.93 (1443)	7.98 (444)	2.57 (143)	0.04 (2)	0.04 (2)	0.02 (1)
L. pneumophila	2,930	4.7×10^{-8}	39.18 (1148)	12.73 (373)	3.89 (114)	0.03 (1)	0.03 (1)	0.03 (1)
S. epidermidis	2,492	5.3×10^{-8}	46.47 (1158)	11.88 (296)	2.89 (72)	0.00 (0)	0.00 (0)	0.00 (0)
S. aureus	2,889	3.2×10^{-8}	40.74 (1177)	10.25 (296)	2.53 (73)	0.00 (0)	0.00 (0)	0.00 (0)
H. influenzae type B	1,704	1.6×10^{-8}	49.35 (841)	16.20 (276)	5.05 (86)	0.06 (1)	0.06 (1)	0.00 (0)

The percentage of similar proteins to the mouse proteome was calculated from the count of similar hits in relation to the total number of proteins of each species. Low stringency = minimum percent coverage of 30% and minimum percent identity of 10%; High stringency = minimum percent coverage of 70% and minimum percent identity of 70%. () = number of proteins with at least one similar mouse protein
